# Associations Between Complex Trauma Exposure in Childhood/Adolescence and Psychopathology in Older Age: The Role of Stress Coping and Coping Self-Perception

**DOI:** 10.1007/s40653-021-00419-0

**Published:** 2021-11-05

**Authors:** Viviane Pfluger, Shauna L. Rohner, Carla M. Eising, Andreas Maercker, Myriam V. Thoma

**Affiliations:** 1 Psychopathology and Clinical Intervention, Institute of Psychology, University of Zurich, Zurich, Switzerland; 2 Dynamics of Healthy Aging”, University Research Priority Program, University of Zurich, Zurich, Switzerland

**Keywords:** Adolescence, Childhood, Complex trauma exposure, Coping self-perception, Mental health, Older adults, Stress coping

## Abstract

Complex trauma exposure in childhood and/or adolescence is common and has repeatedly been linked to mental ill-health across the lifespan. While the correlates of complex trauma and mental health are well-studied in individuals up to middle adulthood, correlates in older adulthood, as well as potential mediators of this relationship, such as stress coping, are insufficiently studied. Therefore, this study aimed to (a) examine the mental health of Swiss older adults affected by complex trauma exposure in childhood and/or adolescence, in comparison to non-affected individuals; and (b) to examine the potential mediating role of coping strategies and coping self-perception. Data from *N* = 257 participants (complex trauma [CT] group: *n* = 161; *M*_age_ = 69.66 years, 48.4% female; non-complex trauma [nCT] group: *n* = 96; *M*_age_ = 72.49 years, 42.7% female) were assessed using self-report questionnaires and a clinical interview. The CT group presented with significantly more current and lifetime mental health disorders, more disadvantageous coping strategies, and significantly lower coping self-perception, compared to the nCT group. Mediation analyses revealed that maladaptive coping and coping self-perception were relevant mediators of the relationship between complex trauma exposure and psychopathology. Results suggest that complex trauma exposure in childhood and/or adolescence can have a lasting impact on mental health in later life and can be negatively associated with stress coping. Findings emphasize the relevance of a lifespan perspective in research and clinical practice for addressing consequences of complex trauma exposure.

## Introduction

Child maltreatment is a common and global phenomenon (Stoltenborgh et al., 2015). Past research has shown that different types of child maltreatment, such as emotional, physical, and sexual abuse, and emotional and physical neglect, often co-exist (e.g., Scher et al., 2004) and are frequently associated with other adverse experiences within the caregiving environment (Green et al., 2010). These particular constellations of multiple interpersonal adversities have previously been referred to as *complex trauma exposure* (Kisiel et al., 2009), which is defined as the experience of traumata that are (a) interpersonal in nature, (b) repeated or chronic, (c) with onset early in life, and (d) occurring within the caregiving system (Cook et al., 2005; Courtois, 2004; Spinazzola et al., 2005). Related terms that partly overlap with this definition and that are also currently used in the literature are *poly-victimization* (Ford et al., 2011), *cumulative adversity* (Seery et al., 2010), or *multiple-type maltreatment* (Higgins, 2004). For the purpose of this paper, the term complex trauma exposure is used to describe exposure to at least two types of child maltreatment in childhood and/or adolescence.

Complex trauma exposure in childhood and/or adolescence has previously been linked to an increased risk for the development of mental health disorders across the lifespan (e.g., Cook et al., 2005; Kisiel et al., 2009). In the *short-term* (i.e., up to adolescence), children and adolescents affected by complex trauma exposure have been shown to present with significantly high(er) rates of a diverse range of mental ill-health outcomes (e.g., Ford et al., 2009; Haahr-Pedersen et al., 2020; Lewis et al., 2021). For example, according to a foster care study by Greeson et al. (2011), a history of complex trauma exposure increased the odds of having at least one clinical mental health diagnosis in youth by 21.3% compared to those who experienced other types of trauma. While less research exists on the *mid-term* (i.e., up to middle-age) mental health sequelae of complex trauma exposure, the few existing studies suggest a higher risk for clinically-relevant psychopathology up to middle adulthood (e.g., Chapman et al., 2004). For instance, in a longitudinal study on the association between child maltreatment and internalizing disorders across adulthood, a history of multiple interpersonal traumata in childhood was associated with an over four times the risk for an internalizing disorder (i.e., depression and anxiety) in adulthood, in comparison to a no or low child maltreatment history (Rapsey et al., 2019). This elevated risk was still detectable 25 years later, which emphasizes the need to also consider the *long-term* (i.e., in older adulthood) mental health sequelae of complex trauma exposure as well as applying a lifespan perspective to this research.

However, there is a lack of research both on the long-term mental health sequelae of complex trauma exposure in older adulthood and up to older age and the mental health consequences from a lifespan perspective (i.e., lifetime disorders). Nonetheless, a few studies have investigated current and lifetime mental health in older survivors of child welfare-related maltreatment, with findings showing an increased risk for a broad range of mental health disorders in survivors of child welfare-related maltreatment when compared to control individuals (i.e., those who were never affected by child welfare practices; e.g., Lueger-Schuster et al., 2018; Thoma et al., 2021). While these studies had a different focus than the current study (i.e., mental health disparities between survivors and controls), it may be assumed that a substantial percentage of the examined individuals were previously exposed to complex trauma constellations, as they showed high prevalence rates for multiple types of child maltreatment (Lueger-Schuster et al., 2018; Thoma et al., 2021). Moreover, a growing body of literature shows child maltreatment-related longer-term correlates with detrimental mental and physical health outcomes (e.g., Bellis et al., 2019; Crouch et al., 2018; Felitti et al., 1998). As such, while there is a lack of research on the long-term mental health sequelae of complex trauma exposure, based on existing related research it can be tentatively assumed that complex trauma exposure may also be linked to an elevated risk for psychopathology both in older adulthood and across the lifespan.

Several theoretical frameworks can be applied to explain how complex trauma exposure may increase the risk for mental ill-health in older adulthood, such as the model of stress sensitization (McLaughlin et al., 2010) or the cumulative advantage/disadvantage theory (Dannefer, 2003). According to these frameworks, (early-life) adversity can have a sensitizing effect upon the affected individuals, which can lower their tolerance (or increase the vulnerability) to future stress experiences and thereby increase the probability for the development of (psycho-)pathology (e.g., Starr et al., 2020; Young et al., 2019). These theoretical assumptions are supported by empirical findings in individuals with a history of (multiple) child maltreatment, such as reporting a stronger tendency to perceive stress more frequently (i.e., heightened perception of minor stressors in everyday life; LoPilato et al., 2020), or an increased risk for further potentially traumatic life events (e.g., Widom et al., 2008). As such, for studies investigating the impact of complex trauma exposure on mental health from a lifespan perspective, it is crucial to consider lifetime traumata as a potentially confounding variable. However, there exist additional aspects that need to be considered when investigating how complex trauma exposure can turn into psychopathology (or not). As such, it is crucial to understand the underlying mechanisms.

One underlying mechanism that may be critically involved in the translation of early-life adversity into an increased stress vulnerability and thus an elevated risk for mental ill-health, is *stress coping*. As such, stress coping refers to cognitive-behavioral strategies initiated as a response to stress and aimed at overcoming the stressful experience. These strategies can either be adaptive or maladaptive, meaning that they result in a successful resolution of a stressor or not (e.g., Folkman & Moskowitz, 2004). More specifically, having predominately maladaptive coping strategies in the repertoire may render an individual more vulnerable towards future stress. Conversely, having a range of adaptive coping strategies could potentially counteract this vulnerability and facilitate positive mental health outcomes (Schneidermann et al., 2005; Taylor & Stanton, 2007). While multiple studies have examined the link between maladaptive stress coping and health in survivors of child maltreatment (e.g., Hager & Runtz, 2012; Van Meter et al., 2020), only one published study has specifically addressed stress coping in survivors of complex trauma exposure. This study by Guerra et al. (2016) linked a history of multiple interpersonal trauma to the frequent use of maladaptive coping strategies based on avoidance and emotional discharge (e.g., worry or self-blame). Moreover, this study also provides empirical evidence for the mediating role of stress coping in the relationship between multiple interpersonal traumata in childhood and internalizing symptoms in adolescence. This emphasizes the relevance of considering stress coping when aiming to understand the mental health sequelae of complex trauma exposure. However, as the study by Guerra et al. (2016) used a young sample (mean age = 14.31 years) and focused on a specific set of coping strategies using a single coping measure, limited knowledge exists on the extent to which stress coping in (older) adults is affected by a history of complex trauma exposure. Furthermore, given that the only existing study was conducted without a control group, it remains unclear whether stress coping differs between individuals as a function of differences in the complexity of their traumatic childhood experiences.

In addition, when investigating stress coping and its association with mental ill-health, it is relevant to not only consider the applied stress coping strategies, but also the subjective perception of an individuals’ stress coping (i.e., efficacy of and satisfaction with stress coping). Following the transactional stress model (Lazarus & Folkman, 1984), stress results from an imbalance between perceived external or internal demands and the perceived personal and social resources to deal with them. As such, the consideration of *coping self-perception* as the cognitive representation of stress coping may be relevant for understanding individual differences in the response to (and potential consequences of) stress. Therefore, to understand how complex trauma exposure can be linked to an elevated risk for psychopathology, the underlying mechanisms of stress coping and coping self-perception should be equally considered. However, to the best of the authors’ knowledge, no study so far has addressed coping self-perception among individuals with a history of complex trauma exposure, nor as a potential translating mechanism for mental health outcomes.

Taken together, research is still largely missing on the long-term sequelae of complex trauma exposure in relation to mental health and stress coping. As the proportion of older individuals in the society is constantly growing (World Health Organization, 2015) and mental health in older age still depicts an understudied area, research on the long-term sequelae of complex trauma exposure is urgently needed. Furthermore, adding a lifespan perspective to this research could help better understand the still insufficiently understood mental health disparities in older age (e.g., Thoma et al., 2021). Focusing on the role of (maladaptive) stress coping in the relationship between the experience of complex trauma and psychopathology is relevant, as stress coping is a potentially modifiable psychological resource (e.g., Allen et al., 2016) and as such lays ground for change processes. Furthermore, it is important to examine the role of stress coping in older individuals, as older age comprises many potentially stressful situations (e.g., retirement, bereavement; Lavretsky & Newhouse, 2012) and tasks (i.e., ‘ego-integrity versus despair,’ see Erikson’s life stage theory (Erikson, 1982). These age-related stressors can put individuals with disadvantageous stress coping (i.e., predominately maladaptive strategies) at an elevated risk for the development or exacerbation of mental health-related problems (Taylor & Stanton, 2007). Additionally, the inclusion of coping self-perception, as a previously neglected aspect of stress coping, offers the opportunity to gain insight into the cognitive representation of the applied stress coping strategies. Such knowledge would be relevant for stress coping interventions. Finally, although complex trauma exposure is typically associated with more complex and long-lasting impacts than non-complex trauma constellations (e.g., Cook et al., 2005; Spinazzola et al., 2005), only a few comparative studies exist (e.g., Lewis et al., 2021; Rapsey et al., 2019). Further comparative evidence is needed on the potential lasting detrimental impact of complex trauma exposure, particularly in older age, as this may help to create better tailored mental health services and interventions for this cohort of particularly vulnerable individuals.

It is therefore the aim of the current study to examine the mental health, stress coping, and coping self-perception of older adults with a history of complex trauma exposure in childhood and/or adolescence, in comparison to a group of older adults with no such history (i.e., single or no trauma exposure during childhood and/or adolescence). The study further aims to determine the potentially mediating role of stress coping and coping self-perception in the relationship between complex trauma history and psychopathology. It is expected that individuals with a history of complex trauma exposure in childhood and/or adolescence will report more current and lifetime mental health disorders, more disadvantageous stress coping strategies, and have lower coping self-perception in comparison to a control group. It is further expected that stress coping and coping self-perception will act as mediators in the relationship between a history of complex trauma exposure and psychopathology.

## Methods

Data were collected between July and December 2019 within a larger research project on differential aging trajectories in the aftermath of early-life adversity (http://www.nrp76.ch/en). The study protocol is in accordance with the Declaration of Helsinki and was approved by the Ethics Committee of the Faculty of Arts and Social Sciences in the University of Zurich (ID: 19.4.3). This study’s mental health data have been previously used by Thoma et al. (2021) to investigate mental health disparities in two different groups (i.e., a risk group of older survivors of compulsory social measures and/or placements [CSMP] compared to a control group).

### Participants

Participants were Swiss adults with a minimum age of 50 years and having Swiss German as native language. Recruitment was conducted via study flyers distributed at various public places (e.g., supermarkets, pharmacies) and places aimed at older citizens (e.g., senior leisure clubs). In addition, flyers were also sent to individuals from a study pool of the affiliated University Research Priority Program, Dynamics of Healthy Aging of the University of Zurich (UZH). As existing research suggest that individuals who were affected by the child welfare practices of the last century had a high risk to be exposed to (complex) traumatic experiences (e.g., Ferguson, 2007), recruitment also aimed to address a particular sample of Swiss individuals, who were affected by CSMP in their childhood and/or adolescence. These individuals were mainly recruited via a contact list provided by the Federal Office of Justice (2020), compiled during the review of the solidarity contribution for those affected by CSMP. Other recruitment methods included word-of-mouth recommendations and contacting publicly active CSMP survivors.

### Procedure

Interested individuals contacted the study team and were screened for the inclusion criteria. If all inclusion criteria were met, two assessment appointments were scheduled (A1 and A2). Each assessment lasted for a maximum of 120 min and was conducted by trained interviewers. Before A1, participants received an information package, the informed consent form, and a set of questionnaires (e.g., demographic information). Upon arrival for A1, the informed consent was signed before starting with the assessment, which consisted of a structured clinical interview to assess current and lifetime mental health disorders. At the end of A1, participants were given a questionnaire package to be completed and returned at A2, covering topics including stress and stress coping. The A2, which took place within one week after A1, collected data on traumatic experiences in childhood and adolescence, lifetime traumata, and various psychological resources and, cognitive as well as functional information. At the end of A2, participants were reimbursed for their participation. The study was conducted at the university or, if preferred, at the participant’s home. Given that the study partly focused on adverse experiences in childhood and/or adolescence, multiple measures were taken to prevent a potential re-traumatization (e.g., Kraemer Tebes et al., 2019) and to minimize potentially occurring major distress: Participants were fully informed about the study content and about the possibility that negative mood/emotions could be elicited by participating in the study. Participants could take breaks at any time during the assessment or withdraw from participation without negative consequences. Trauma-related questions were part of the face-to-face assessments, only, during which the interviewers (trained for dealing with traumatized individuals) could immediately respond to major emotional distress. Finally, participants were provided with a list of contact points for crises intervention and psychological counselling.

### Measures

#### Complex Trauma Exposure

A history of complex trauma exposure in childhood and/or adolescence was assessed with the German version of the Childhood Trauma Questionnaire (CTQ; Bernstein & Fink, 1998; Gast et al., 2001). The CTQ is a self-report questionnaire assessing emotional, physical, and sexual abuse, as well as emotional and physical neglect. The five childhood trauma-related subscales consist of five items each, with items rated on a 5-point Likert scale ranging from 1 (*not at all*) to 5 (*very often*). Potential subscale scores range from 5 to 25, with higher scores indicating more childhood trauma. The severity of each trauma type was calculated as proposed by Häuser et al. (2011), meaning that each trauma type was considered present if the level ‘moderate to severe’ or higher was indicated. As proposed by Kisiel et al., (2009), to build a grouping variable for a history of complex trauma exposure, complex trauma exposure was operationalized as the presence of two or more interpersonal traumatic experiences in childhood and/or adolescence at an actionable level (i.e., severity ratings of moderate to extreme). In the present study, the internal consistency of all five subscales was high (emotional abuse $\boldsymbol{\alpha }$ = 0.83; physical abuse $\boldsymbol{\alpha }$ = 0.83; sexual abuse $\boldsymbol{\alpha }$ = 0.96; emotional neglect $\boldsymbol{\alpha }$ = 0.88; physical neglect $\boldsymbol{\alpha }$ = 0.78).

#### Stress Coping and Coping Self-Perception

Stress coping strategies were measured with the German version of the Questionnaire for Individual Coping (INCOPE; Bodenmann, 2000). The INCOPE is a self-report questionnaire assessing maladaptive and adaptive individual coping strategies with two subscales. The subscales consist of 10 and 11 items, respectively, with items rated on a 5-point Likert scale ranging from 1 (*never*) to 5 (*usually*). Potential subscale scores range from 10 to 50 for maladaptive coping and 11 to 55 for adaptive coping, with higher scores indicating more frequent use of the corresponding coping strategies in everyday life. In the present study, the internal consistency was acceptable for the maladaptive subscale ($\boldsymbol\alpha$ = 0.68) and good for the adaptive subscale ($\boldsymbol{\alpha }$ = 0.79).

For coping self-perception, two items from the INCOPE were used: one item on ‘coping efficacy’ (‘*My way of dealing with stress is usually effective*’) and one item on ‘satisfaction with coping’ (‘*I am satisfied with the way I deal with stress’*). Both items again being rated on a 5-point Likert scale ranging from 1 (*never*) to 5 (*usually*). Scores from the two items were summed to create a composite score, with a potential range of 2 to 10; and higher scores indicating a better coping self-perception. The internal consistency for this composite score was good ($\boldsymbol{\alpha }$ = 0.80).

#### Psychopathology

To assess psychopathology, the German structured clinical interview for diagnosing mental health disorders was used (DIPS; Margraf et al., 2017a, b), which allows for the diagnosis of current and lifetime mental health disorders according to the DSM-5. In addition to the individual level of current and lifetime psychopathology, an index score was calculated (i.e., number of current and lifetime mental health diagnoses). The following mental health disorders were assessed in the current study: anxiety disorders, affective disorders, obsessive–compulsive disorders, posttraumatic stress disorder, somatic stress disorders, sleep–wake disorders, and disorders related to psychotropic substance and dependence behaviors. The potential index score of psychopathology ranged from 0 to 40, with higher scores indicating more mental health disorders.

#### Lifetime Trauma 

Lifetime trauma was measured using a list of 18 potentially traumatic experiences across the lifespan, which were included in the PTSD section of the DIPS (Margraf et al., 2017a, b). For each of the 18 traumatic experiences (e.g., sexual violence in adulthood, serious accident), participants indicated whether they had experienced it (yes = 1) or not (no = 0). Based on these answers, the total number of reported lifetime traumata was calculated (i.e., number of different traumatic experiences; range: 0 to 18) and used as a control variable in the analyses.

### Data Analysis

All analyses were performed using IBM Statistical Package for Social Sciences (SPSS) version 26 (IBM Corp, 2020). Missing values (< 1% for each questionnaire) were replaced using the Expectation–Maximization algorithm with 25 iterations (Dempster et al., 1977), as Little’s missing completely at random (MCAR) test suggested that the values were MCAR.

To test whether individuals with and without a complex trauma exposure history differ in their level of psychopathology, a one-way ANCOVA was conducted with complex trauma exposure (no/yes) as the independent variable and psychopathology as the dependent variable, whilst controlling for the number of lifetime traumata. In addition, group differences in the included mental health disorders (both current and lifetime) were tested using Pearson’s Chi-squared test. To test whether individuals with and without a complex trauma exposure history differ in the way they cope with stress (maladaptive and adaptive stress coping) and how they perceive their stress coping (coping self-perception); three one-way analysis of covariance (ANCOVA) were conducted with complex trauma exposure (no/yes) as the independent variable and maladaptive coping, adaptive coping, and coping self-perception as respective dependent variables. The number of lifetime trauma was included as a covariate in the analyses. Finally, to investigate whether the relationship between complex trauma exposure and psychopathology was mediated by stress coping and coping self-perception, a parallel mediation analysis (model 4) was performed using the PROCESS version 3.0 macro for SPSS (Hayes, 2013). This uses ordinary least squares regression, yielding unstandardized path coefficients for total, direct, and indirect effects. Bootstrapping with 5000 samples was employed to compute the confidence intervals and inferential statistics. Parallel mediation analysis was expected to be unproblematic as the two potential mediators were only moderately correlated (*r* = -0.265). Effects were deemed significant when the confidence interval did not include zero. As maladaptive stress coping and coping self-perception were significantly related to a history of complex trauma exposure, these two variables could be included as potential mediators. As adaptive stress coping was not significantly related to a history of complex trauma exposure, it was not examined as a mediator.

## Results

### Sample Characteristics

The study sample consisted of *N* = 257 participants (mean age = 70.72 years, *SD* = 11.08), with *n* = 161 participants (62.2%) meeting the criteria for complex trauma exposure (CT group) and *n* = 96 participants (37.4%) not meeting these criteria (non-complex trauma exposure; nCT group). The two groups were comparable with regard to sex, relationship, and employment status; but differed significantly (*p* < 0.05) with respect to age, education, and income (see Table 1 for sample characteristics).

Individuals in the CT group experienced on average 3.55 (*SD* = 1.03, range 2–5) potentially traumatic experiences during their childhood and/or adolescence, with emotional neglect (93.8%) being the most prevalent trauma type, followed by physical neglect (85.7%), emotional abuse (59.0%), sexual abuse (58.6%), and physical abuse (58.4%). Table 2 summarizes the combined number of childhood and/or adolescence trauma in both groups.

**Table 1:** Sample Characteristics

	Total sample (*N* = 257)	CT (*n* = 161)	nCT (*n* = 96)	Group comparison
Age (*M, SD;* range = 49–95)	70.72 (11.08)	69.66 (11.34)	72.49 (10.46)	*t*(255) = 1.992, *p* = .047
Sex (female) (%)	46.3	48.4	42.7	X^2^ = 0.797*, p* = .372
Relationship status (%)				X^2^ = 10.661*, p* = .059
Single	12.5	13.0	11.5	
In a relationship	11.3	13.0	8.3	
Married	41.2	36.6	49.0	
Separated	1.9	3.1	0	
Divorced	20.2	23.6	14.6	
Widowed	12.8	10.6	16.7	
Employment status (%)				X^2^ = 5.044*, p* = .283
Employed	21.4	24.8	15.6	
Unemployed	2.7	3.1	2.1	
Retired/pension	58.0	53.4	65.6	
Voluntary work	11.7	10.6	13.5	
Highest level of education (%)				X^2^ = 19.849*, p* = .006
No education	2.3	3.1	1.0	
Primary school	3.9	5.0	2.1	
Upper secondary school	10.5	13.7	5.2	
Secondary/High school	2.3	1.9	3.1	
Vocational job training	39.3	42.9	33.3	
Higher professional training	14.8	14.9	14.6	
University level	21.8	14.3	34.4	
Income (per month) (%)				X^2^ = 26.612*, p* $<$ .001
< 2001 Swiss Francs	15.2	20.5	6.3	
2001 – 3330 Swiss Francs	19.8	25.5	10.4	
3331 – 4670 Swiss Francs	16.7	16.8	16.7	
> 4670 Swiss Francs	46.7	35.4	65.6	

*CT* complex trauma group, *nCT* no complex trauma group, *M* mean, *SD* standard deviation, *X*^*2*^ Pearson’s Chi-squared test, *t* two-sided t-test comparing complex trauma group with no complex trauma group, *p* *p*-value

**Table 2:** Number of Types of Childhood/Adolescent Trauma Separately for Individuals With and Without Complex Trauma Exposure History

	CT (*n* = 161)	nCT (*n* = 96)
CTQ	*n* (%)	*n* (%)
No traumatic experience		57 (59.4)
One type of trauma		39 (40.6)
Two types of trauma	28 (17.4)	
Three types of trauma	53 (32.9)	
Four types of trauma	43 (26.7)	
Five types of trauma	37 (23.0)	

*CTQ* Childhood Trauma Questionnaire, *CT* Complex trauma group, *nCT* No complex trauma group

### Group Differences in Current and Lifetime Psychopathology

Table 3 displays detailed information on the current and lifetime psychopathology for the total sample, as well as separately for the CT and nCT groups. Across both groups, 47.1% of the participants presented with a current mental health disorder (CT group: 57.1%; nCT group: 30.2%); 80.9% presented with a lifetime mental health disorder (CT group: 85.1%; nCT group: 74.0%); and 17.9% reported never having experienced any of the assessed mental health disorders (CT group: 14.3%; nCT group: 24.0%). The most common lifetime disorders were major depression (40.4%), PTSD (26.7%), and dysthymia (21.7%) in the CT group; and major depression (26.0%), dysthymia (9.4%), and panic disorder (8.3%) in the nCT group. With regard to current disorders, both groups presented with a highest prevalence of internalizing disorders, such as specific phobia (CT group: 13.7%; nCT group 7.3%) and separation anxiety (CT group: 13.0%; nCT group 4.2%).

**Table 3:** Mental Health Disorders (Diagnoses) and Group Comparison

	Groups							Group comparison			
Diagnosis, *n* (%)	CT				nCT			Current		Lifetime	
	Current		Lifetime		Current		Lifetime	$\mathrm{\rm X}$^2^	*p*	$\mathrm{\rm X}$^*2*^	*p*
Anxiety disorders											
Separation anxiety	21 (13.0)		24 (14.9)		4 (4.2)		6 (6.3)	7.129	**	4.501	*
Panic disorder	5 (3.1)		16 (9.9)		3 (3.1)		8 (8.3)	0.001	1^a^	0.206	.650
Agoraphobia	14 (8.7)		13 (8.1)		1 (1.0)		3 (3.1)	6.508	*	2.589	.108
Social phobia	19 (11.8)		25 (15.5)		3 (3.1)		7 (7.3)	5.904	*	3.868	*
Specific phobia	22 (13.7)		20 (12.4)		7 (7.3)		4 (4.2)	4.697	*	4.958	*
Generalized anxiety	18 (11.2)		17 (10.6)		4 (4.2)		7 (7.3)	3.877	*	0.805	.370
Bipolar disorders											
Bipolar disorder I	1 (0.6)		4 (2.5)		0		1 (1.0)	0.605	1^a^	0.674	.653^a^
Bipolar disorder II	1 (0.6)		1 (0.6)		0		0	0.605	1^a^	0.605	1^a^
Depressive disorders											
Dysthymia	16 (9.9)		35 (21.7)		2 (2.1)		9 (9.4)	5.801	*	6.690	**
Major depression	14 (8.7)		65 (40.4)		1 (1.0)		25 (26.0)	6.508	*	5.782	*
Obsessive–compulsive disorders											
Compulsive thoughts	3 (1.9)		4 (2.5)		0		0	1.830	.293^a^	2.449	.300^a^
Compulsive actions	6 (3.7)		6 (3.7)		0		0	3.704	.086^a^	3.704	.086^a^
Trauma and stress related disorders											
Posttraumatic stress disorder	19 (11.8)		43 (26.7)		1 (1.0)		6 (6.3)	9.847	**	16.699	***
Somatic stress disorders											
Somatic disorder	15 (9.3)		17 (10.6)		3 (3.1)		5 (5.2)	3.623	.057	2.274	.132
Hypochondria	1 (0.6)		1 (0.6)		1 (1.0)		1 (1.0)	0.132	1^a^	0.132	1^a^
Sleep–wake disorders											
Insomnia	23 (14.3)		27 (16.8)		11 (11.5)		8 (8.3)	0.631	.427	3.321	.068
Hypersomnia	3 (1.9)		2 (1.2)		2 (2.1)		3 (3.1)	0.013	1^a^	1.091	.368^a^
Disorders related to psychotropic substances and dependence behaviors											
Alcohol consumption disorders 0			3 (1.9)		0		1 (1.0)			0.275	1^a^
Smoking	40 (24.8)		91 (56.5)		8 (8.3)		47 (49.0)	11.095	***	2.277	.131
Drugs	2 (1.2)		8 (5.0)		0		2 (2.1)	1.215	.529^a^	1.375	.328^a^

*CT* complex trauma group, *nCT* no complex trauma group, *X*^*2*^ Pearson’s Chi-squared test

* *p* < .05; ** *p* < .01; *** *p* $$\le$$ .001

^*a*^ Fisher’s exact test,* p* *p*-value

Regarding the overall level of psychopathology, the one-way ANCOVA showed a significantly higher mean score of psychopathology in the CT group (*M* = 4.15, *SD* = 4.05) compared to the nCT group (*M* = 2.02, *SD* = 2.11; *F*(1,253) = 14.815, *p* < 0.001, η_p_^2^ = 0.055). The covariate ‘number of lifetime trauma’ also showed a significant positive effect on psychopathology (*F*(1,253) = 13.660, *p* < 0.001, η_p_^2^ = 0.052). These results suggest that having a history of complex trauma exposure, as well as a higher number of lifetime trauma, was associated with higher levels of current and lifetime psychopathology.

### Group Differences in Stress Coping and Coping Self-Perception

Results of the one-way ANCOVA showed a significantly higher mean score for maladaptive stress coping in the CT group (*M* = 24.94, *SD* = 5.91), compared to the nCT group (*M* = 22.51, *SD* = 4.52; *F*(1, 254) = 8.445, *p* < 0.001, η_p_^2^ = 0.032). While the mean score for adaptive stress coping was higher in the nCT group (*M* = 35.30, *SD* = 7.25) than in the CT group (*M* = 33.95, *SD* = 9.38), this effect was not significant (*F*(1,254) = 2.810, *p* = 0.095, η_p_^2^ = 0.011). Results showed a significantly lower mean score for coping self-perception in the CT group (*M* = 6.83, *SD* = 2.06) compared to the nCT group (*M* = 7.51, *SD* = 1.87; *F*(1, 254) = 7.459, *p* = 0.007, η_p_^2^ = 0.029). The covariate ‘number of lifetime trauma’, did not have a significant effect on any of the three dependent variables (*p* > 0.20).

### Association Between Complex Trauma Exposure and Psychopathology

**Fig. 1:**
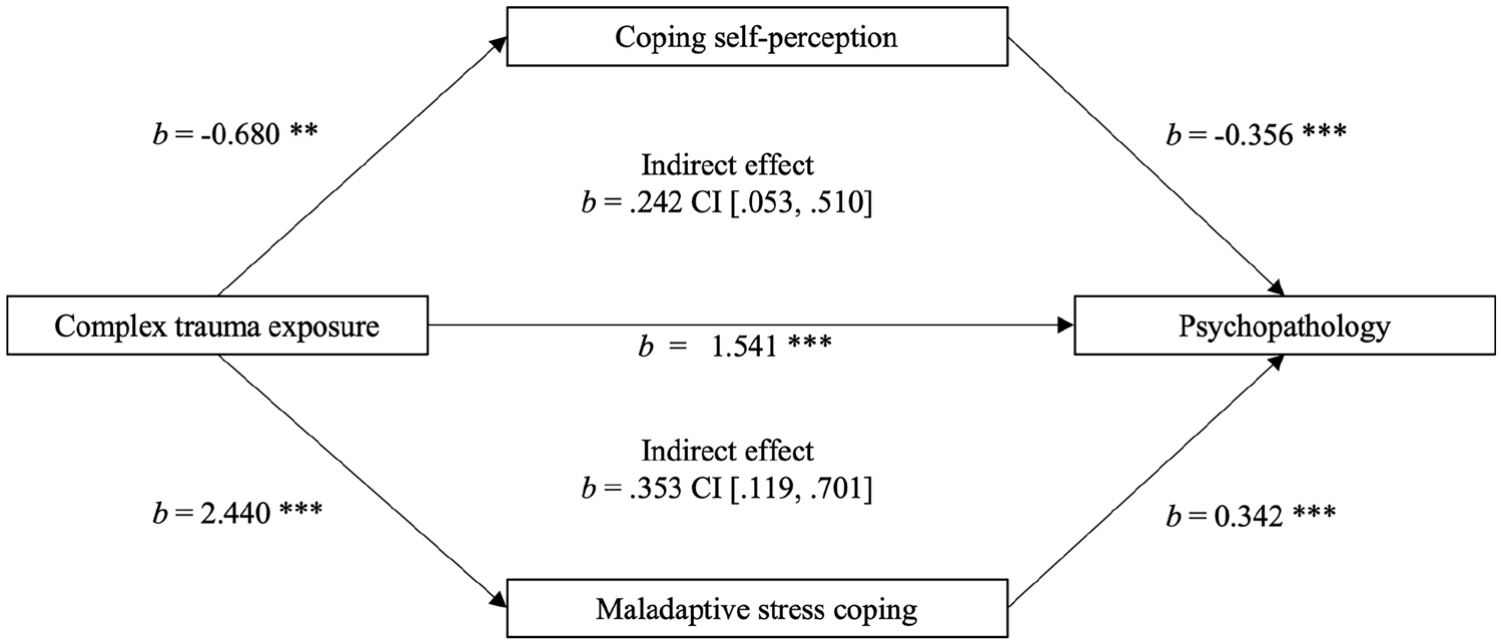
Parallel Mediation Model of the Relationship Between Complex Trauma Exposure History (Predictor) and Overall Psychopathology (Outcome Variable), Significantly Mediated by Maladaptive Stress Coping and Coping Self-Perception (Mediators)

The parallel mediation analysis revealed a partial mediation of the relationship between complex trauma exposure and psychopathology by maladaptive stress coping and coping self-perception. Initially, a significant total effect was observed between complex trauma and psychopathology (*b* = 2.440, *t* = 3.470, *p* = 0.001), explaining 21.3% of the variance in psychopathology. When maladaptive stress coping and coping self-perception were included as mediators in the model, a significant direct effect emerged for complex trauma exposure and psychopathology, explaining a greater percentage (28.8%) of the variance. A significant indirect effect via both maladaptive stress coping (*b* = 0.353, 95% CI [0.119, 0.701], Z = 2.497, *p* = 0.013) and coping self-perception (*b* = 0.242, 95% CI [0.053, 0.510], Z = 2.023, *p* = 0.043) was also observed, indicating that the relationship between complex trauma exposure and psychopathology levels can be partly explained through higher levels of maladaptive stress coping and lower levels of coping self-perception. See Fig. 1 for the mediation model.

## Discussion

This study aimed to examine current and lifetime mental health, stress coping, and coping self-perception in older adults with and without a history of complex trauma exposure in childhood and/or adolescence. It further aimed to determine the role of stress coping and coping self-perception in the relationship between complex trauma exposure in childhood and/or adolescence and psychopathology later in life. Results showed that individuals affected by complex trauma exposure had a higher mental health burden across the lifespan and in older age. Affected individuals also presented with more disadvantageous stress coping strategies and a lower coping self-perception compared to non-affected individuals. Maladaptive stress coping and coping self-perception acted as significant mediators in the relationship between complex trauma exposure history (no/yes) and psychopathology, suggesting that differences in stress coping and coping self-perception between the two groups may be associated with differences in mental health.

More than half of the investigated sample of older Swiss individuals met the criteria for a complex trauma exposure history. As a substantial percentage of these individuals (73.3%) were affected by CSMP, this high number lends support to the notion that child maltreatment, particularly when it happens within the context of child welfare-related practices, is often not an isolated aversive experience (Stoltenborgh et al., 2015). As such, this finding emphasizes the need of further research addressing child maltreatment both within the context of child welfare-related practices and other high-risk contexts from a complex trauma exposure perspective.

In the current study, individuals with a history of complex trauma exposure in childhood and/or adolescence reported a high(er) mental health burden during their life. This was indicated by meaningfully high(er) prevalence rates for a broad range of current and lifetime mental health disorders, as well as a significantly higher psychopathology index score, corroborating findings on the detrimental long-term correlates of child maltreatment (see, e.g., review by Bellis et al., 2019). The higher lifetime prevalence rates for various anxiety and depressive disorders and for PTSD align with previous findings derived from younger samples (e.g., Greeson et al., 2011; Kisiel et al., 2009; Lewis et al., 2021; Rapsey et al., 2019). With regard to current mental health, significantly higher prevalence rates for various anxiety and depressive disorders, PTSD, and smoking were found for individuals with a history of complex trauma exposure. These findings were expected on the basis of previous studies with younger samples (e.g., Greeson et al., 2011; Lewis et al., 2021; Rapsey et al., 2019) and further corroborate the notion that complex trauma exposure in childhood and/or adolescence can depict a lifelong burden that can reach into later life. Regarding the related study by Thoma et al. (2021) on mental health disparities in survivors of CSMP and controls, the groups from these two studies partially overlap and the results go into the same direction in the understanding of child maltreatment. Taken together, the overall findings suggest a high mental health burden of older individuals with a CSMP history, which becomes even more apparent when complex trauma constellations are considered. Despite the findings for a high mental health burden across the lifespan following complex trauma exposure, it is also possible that individuals show resilient outcomes/trajectories after these highly adverse experiences and develop resilient outcomes (see, e.g., Masten, 2001; Ungar, 2013; Van Breda, 2018). As such, future research investigating complex trauma exposure sequelae from a salutogenic perspective would be needed.

In contrast to previous findings (e.g., Cook et al., 2005; Spinazzola et al., 2005), individuals with a history of complex trauma exposure presented with comparably low prevalence rates for substance abuse, such as alcohol abuse (lifetime: 1.9%; current: 0% in the present study vs. 18% at the age of 18 years as reported in the study by Lewis et al., 2021). Potential explanations for these differences may be that the older generation tends to under-report alcohol consumption due to fear of social stigmatization or that former high users have been affected by premature death due to alcohol-related illness (e.g., Blow & Barry, 2012). In contrast, the high number of current smokers within the group of complex trauma survivors (24.8%) is consistent with previous findings (e.g., 25% at the age of 18 years as reported in the study by Lewis et al., 2021). As smoking can also be considered as a form of stress coping (e.g., Bodenmann, 2000), it may be that survivors of complex trauma exposure use smoking as a means of dealing with the high emotional burden and the potential manifold consequences (e.g., problems in the regulation of emotions; Cook et al., 2005) associated with their early experiences.

Analyses of stress coping strategies across both groups revealed a more disadvantageous strategy for individuals affected by complex trauma exposure. This means that affected individuals used significantly more maladaptive coping strategies and had the tendency to use less adaptive coping strategies compared to non-affected individuals. These differences may partly be explained by the assumption that children and adolescents who grow up in stable and safe environments have the opportunity to develop adaptive coping strategies, for instance by having role models who exemplify a successful way to cope with stress. In contrast, individuals affected by complex trauma exposure may be more likely to encounter conditions characterized by a lack of stability and safety and may therefore focus more on survival in the course of development (Kinniburgh et al., 2005).This focus may lead to the development of maladaptive coping strategies and may impede them from accumulating coping resources (e.g., social support). Maladaptive coping strategies, such as avoidance behavior or self-harm, could be helpful to resolve the immediate threat and its short-term negative consequences, but may not help to process the traumatic experience appropriately or to facilitate coping in the long-term with stressful situations later in life (Courtois & Ford, 2016; Mahoney et al., 2019). Another possible explanation for the finding that complex trauma exposure is associated with high maladaptive stress coping could be the (chronic) problems in various areas of self-regulation (e.g. emotion regulation) that may arise from complex trauma exposure (e.g., Spinazzola et al., 2005). These problems may lead to difficulties in building up (more) adaptive coping strategies later in life and may also perpetuate the sustained use of maladaptive coping strategies (Kinniburgh et al., 2005). To examine this interpretation, further studies applying a multiple pathway structural equation modelling approach are needed. Such an approach would allow for the estimation of interdependencies between multiple pathways and to selectively investigate if pathways vary between groups (Kline, 2015). This could bring further insight into the relevance of stress coping in the relationship between complex trauma exposure and the development of psychopathology later in life.

In the current study, affected individuals also presented with significantly lower levels of coping self-perception, suggesting that on average, affected individuals perceive their stress coping as less effective and satisfying than non-affected individuals. This novel finding represents a promising aspect for interventions (e.g., as part of the pre- and post-evaluation of stress coping interventions) and should be examined in more detail in future research. With regard to the use for such evaluation processes, given this study’s cross-sectional design, future studies should aim to investigate coping self-perception from a longitudinal perspective to evaluate its stability over time.

Maladaptive stress coping and coping self-perception were found to partially mediate the relationship between complex trauma exposure history and mental health, with maladaptive stress coping being the slightly more substantive mediator. This finding is in line with previous work by Guerra et al., (2016), which highlighted the relevance of maladaptive stress coping in the understanding of the mental health sequelae of multiple interpersonal traumatic experiences in a younger sample. Moreover, given that in the current study the mediation analysis used the grouping variable (i.e., complex trauma exposure no/yes) as the independent variable, the results suggest that differences in maladaptive stress coping and coping self-perception between affected and non-affected individuals may be associated with differences in mental health. Thus, stress coping and coping self-perception may constitute relevant factors in the understanding of mental health disparities in older age, which is still insufficiently understood (e.g., Thoma et al., 2021). However, in the present study the groups did not significantly differ with respect to adaptive stress coping and so this potential (protective) mediating association could not be investigated (see, e.g., Maercker et al., 2016). For a more complete picture of how stress coping is involved as a mechanism in the relationship between complex trauma exposure and psychopathology, further investigations addressing both adaptive and maladaptive stress coping are needed.

The current study extends existing literature in several ways. First, besides the existing studies on complex posttraumatic stress disorder (e.g., Krammer et al., 2016), the current study is the first to address the long-term (i.e., in later life) mental health sequelae of complex trauma exposure in childhood and/or adolescence. This study also contributes to existing knowledge by using a broad range of current and lifetime mental health disorders. Second, the current study expands on the few existing comparative studies on the mental health of individuals with and without a history of complex trauma exposure (e.g., Greeson et al., 2011; Lewis et al., 2021) by comparing two groups of older adults. The inclusion of a control group is not yet common practice in studies investigating the long-term impact of child maltreatment (see, e.g., Carr et al., 2020). However, the use of a control group is essential for the adequate integration of the findings within the existing trauma research and to better understand the particular vulnerability of individuals with a complex trauma exposure history compared to those without complex trauma exposure. This knowledge is key for both prevention and intervention. Third, this study also adds a novel contribution to the current literature on stress coping by examining the cognitive representation of stress coping in the form of coping self-perception. Given that the motivation for change in the individual approach to stress coping might substantially arise from the satisfaction with and the perceived efficacy of ones’ stress coping (see, e.g., transtheoretical model of health behavior change; Prochaska & Velicer, 1997), this research could be relevant with regard to stress coping interventions, as mentioned above.

There are several limitations in the current study that must be taken into consideration when interpreting the findings. First, given the cross-sectional, retrospective study design, no causal conclusions can be drawn. In addition, groups were assigned according to retrospectively assessed data on child maltreatment. These data could have been affected by a memory recall and retrieval bias (Sheikh, 2018). Third, given the advanced age of the study sample and the known health impeding effects of child maltreatment (Clemens et al., 2018), recruitment could have been affected by a survivor bias. This could have led to an underestimation of the proportion meeting the criteria for complex trauma exposure as well as of the mental health differences between the two groups. However, given that the study operated with a non-blinded study purpose, which in turn might have led to greater self-selection of participants with higher rates of child maltreatment experiences, the found proportion of complex trauma exposure might nevertheless represent a rather realistic estimation. As such, it is also comparable to previous prevalence rates within the child welfare context (70.4%; Greeson et al., 2011). Lastly, given that the study used a heterogeneous set of individuals with no or single trauma history in childhood and/or adolescence as a control group, no definitive statements can be made about differences in the long-term sequelae of single and complex trauma exposure. Future studies should aim to make this distinction in order to evaluate if the particular short-term vulnerability of complex trauma exposure survivors compared to single trauma survivors outlined by Greeson et al. (2011) remains present into older age. Nevertheless, the current study found meaningful group differences with respect to mental health, expanding the current findings from the existing comparative studies on the short-term (Lewis et al., 2021) and mid-term (Rapsey et al., 2019) mental health sequelae of complex trauma exposure.

Complex trauma exposure is an extremely detrimental experience that has a high potential to affect mental health into later life. The present study is the first to specifically examine the long-term mental health sequelae in older adulthood, providing evidence of a long-term mental health burden in Swiss older adults with a history of complex trauma exposure in childhood and/or adolescence. As such, this study lays the groundwork for future research on the long-term sequelae of complex trauma exposure. In addition, findings on stress coping and coping self-perception contribute to a better understanding of potential impairment later in life. Study findings also highlight the importance of research explicitly focused on individuals with a complex trauma exposure history, so that these highly vulnerable individuals are not obscured in the steadily growing literature on the (lifetime) impact of child maltreatment.

## Data Availability

Due to the sensitive nature of the data, the data cannot be published on a public data repository. The raw data will instead be held in the university archives in accordance with the ethical regulations.
